# Redescription of the *Lutztrema* (*Lutziella*) *microacetabularae* Rohde, 1966 trematode of the Family Dicrocoelidae (Looss, 1899)

**Published:** 2013

**Authors:** Noor UN-NISA, Wali KHAN, Aly KHAN

**Affiliations:** 1 Vertebrate Pest Control Institute (VPCI), Southern Zone-Agricultural Research Centre (SARC), Pakistan Agricultural Research Council (PARC), old Block 9 &10 Karachi University Campus Karachi, Pakistan; 2 Crop Diseases Research Institute (C.D.R.I), Southern Zone-Agricultural Research Centre (SARC), Pakistan Agricultural Research Council (PARC), SARC, Karachi University Campus, Karachi, Pakistan

**Keywords:** Rattusrattus, Small intestine, Trematode, Pakistan

## Abstract

**Background:**

A known species *Lutztrema (Lutziella) microacetabularae* Rhode, 1966 is being described for the first time from Pakistan. This species is characterized by having body long and slender, oral sucker subterminal, acetabulum smaller than oral sucker lying in anterior third of the body, pharynx small, esophagus prominent which become gradually wider and bifurcates in to two rudimentary caeca. Testes symmetrical at the level of posterior margin of acetabulum separated by uterine coils, cirrus pouch median, pre-acetabular, genital opening some distance behind pharynx. Receptaculumsemin is behind ovary. Ovary submedian, post-testicular, Laurer's canal present. Vitellaria lateral from the level of testes to a short distance behind middle of the body. Uterus occupies most of the hind body, eggs small, oval, numerous. It is being reported from the rat (*Rattusrattus* L.) from Swat.

## Introduction

Studies on helminth parasites of vertebrates have been done in the world but in Pakistan such type of studies have been confined and restricted to targeted animal populations. Mostly these studies have been undertaken on fishes (1, 2) and birds (3–5) while no such work has been carried out on rodents of Swat, Pakistan.

The present study was conducted in March 2011. During this study 11 trematodes of the genus *Lutziella* Rohde, 1966 (6) were recovered from the small intestine of *Rattusrattus* which are being described in detail.

## Materials and Methods

Live rats (*Rattusrattus*L.) were collected from the maize field of Miandam, Swat and brought to VPCI, SARC, Karachi. The rats were anesthetized and dissected for the presence of helminth parasites. During the examination eleven trematodes belonging to the genus *Lutziella* Rohde, 1966 were recovered from the small intestine of the host. Specimens were fixed in FAA solution under slight cover glass pressure, stained in Mayer's carmalum, dehydrated in graded series of ethanol, cleared in clove oil and xylol and mounted in Canada balsam. Measurements are in millimeters. Specimens are in the possession of (W.K).

**Table:** 

Host:	*Rattusrattus* (L.)
Location:	Small intestine
Locality:	Maize fields, Miandam, Swat, Pakistan
No. of specimens recovered:	Eleven from two host
No. of host examined:	130

### Lutztrema(Lutziella)microacetabulareRohde, 1966

(Figs. 1–2)

**Fig. 1:**
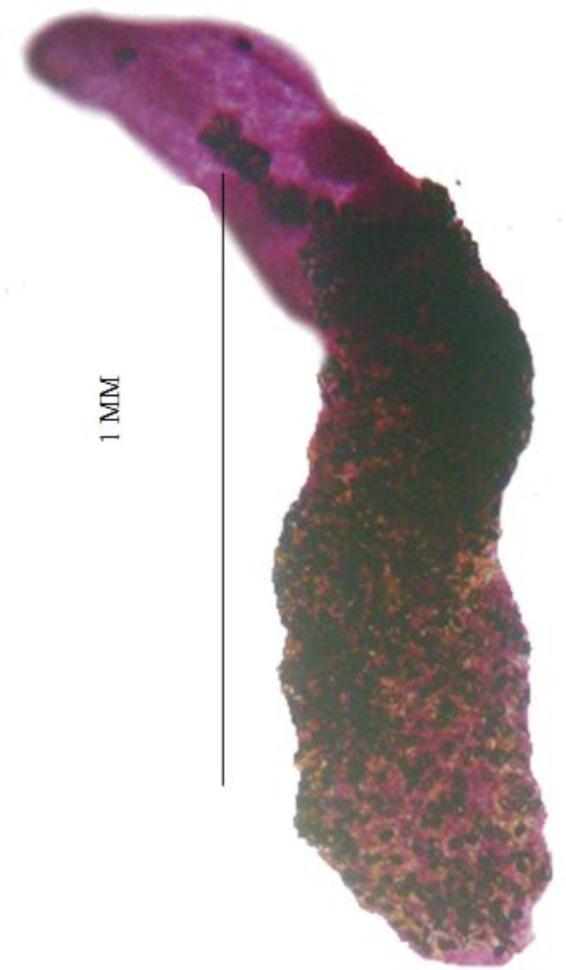
Photomicrograph of Lutztrema (*Lutziella*) *microacetabularae*, holotype

**Fig. 2:**
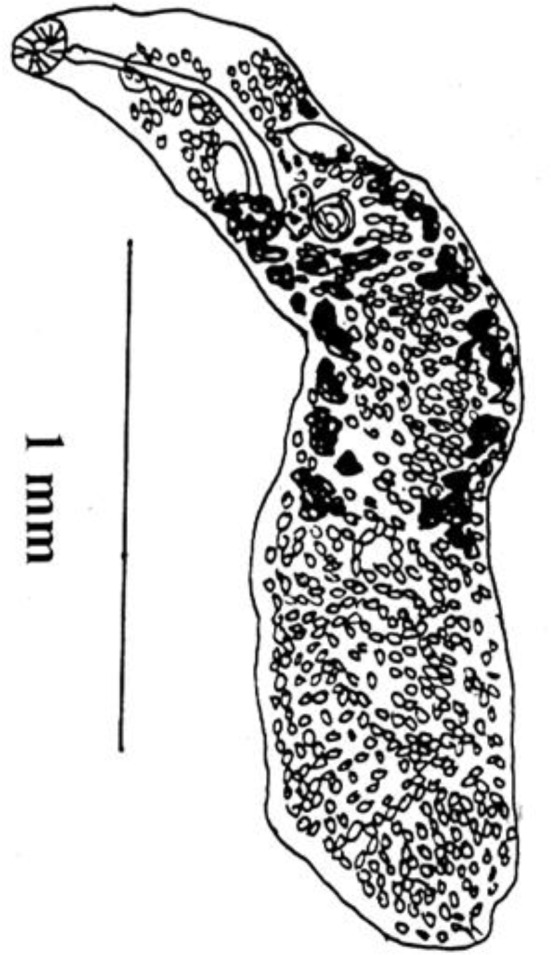
*Lutztrema (Lutziella) microacetabularae*, Holotype entire

## Case

Body long and slender measuring 1.64-2.80 by 0.68-0.88, Oral sucker subterminal, measuring 0.12-0.24 by 0.08-0.12. Acetabulum smaller than oral sucker measuring 0.10-0.15 by 0.075-0.10 lying in anterior third of the body.Pharynx small measuring 0.08- 0.12 by 0.04- 0.08.Eosophagus prominent measuring 0.075 – 0.15 by 0.03-0.10 which become gradually wider and bifurcates in to two rudimentary caeca. Testes symmetrical at the level of posterior margin of acetabulum separated by uterine coils. The left measuring 0.24 – 0.27 by 0.15 – 0.21, while the right measuring 0.22 – 0.25 by 0.10 – 0.19. Cirrus pouch median, preacetabular, genital opening some distance behind pharynx. Cirrus pouch measures 0.09- 0.15 by 0.30 –0.060.Receptaculumsem-inislocated behindthe ovary. Ovary submedian, post-testicular measuring 0.08 – 0.20 by 0.04- 0.024. Laurer's canal present. Vitellaria lateral from the level of testes to a short distance behind middle of the body. Uterus occupies most of the hind body, eggs small, oval, numerous measuring 0.022 – 0.41 by 0.015 – 0.019.

## Discussion

As indicated by the structure of caeca, the new form is among all Dicrocoeliidae, most closely related to *Lutztrema*, Travassos, 1941, but differs from *Lutztrema*in the testes are oblique while in present specimen they are symmetrical; moreover in *Lutztrema* acetabulum is larger than oral sucker while in present genus it is smaller than oral sucker. In *Lutztrema* the vitellaria are post ovarian, while in the present specimens it extends in to the space in front of the ovary. Due to the combinations of all these characters the species belong to the genus *Lutziella* Rohde, 1966, although there are small difference in the size of pharynx and testes, the rest characters are in accordance from the host *Myotismystacinus* (Kahl) from Jandabaik, Pahang Malaya and thus is regarded the same from a new host and locality.

## References

[1] Khan A, Bilqees FM (1990). Fourtrematodes inclu-ding a new species from freshwater fishes of Sindh, Pakistan. Proceedings of Parasitology..

[2] Bilqees FM, Ghazi RR, Khan A, Khatoon N (2004). *Parapolylekithumkarachiensisn*. gen., n.sp.(Digen-ea: Allocreadiidae: Allocreadiinae) from the fish *Cybiumguttatum* of Karachi coast. Turkiye Parazitoloji Dergis..

[3] Khan A, Das SN, Ghazi RR, Noor Un Nisa (2009). A new species of trematode genus *Schwatzitrema Perez vigueras*, 1941 (Strigeidae) from a bird (Cattle egret, *Bubulcus ibis* L.) in Sindh, Pakistan. Int J Biol Biotech.

[4] Khan A, Ghazi RR (2011). *Nephrostomusoderolala-nensisn*.sp., (Trematoda: Digenea) in Cattle egret (*Bubulcus ibis* L.) from Sindh, Pakistan. Int J Biol Biotech..

[5] Das SN, Ghazi RR (2012). *Apharyngostrigeamegavatas*-p.n. (Trematoda: Strigeidae, Railliet, 1919) from a new host *Egrettaalba* (Linneaus) in Sindh, Pakistan. Proceedings of Parasitology..

[6] Rohde K (1966). On the trematode genus *Lutztrema* Travassos, 1941 and *Anchitrema* Looss, 1899 from Malayan Bats, with discussion of Allometric growth in Helminths. The Helmi-nthological Society of Washington..

